# An updated systematic review of interventions to increase awareness of mental health and well-being in athletes, coaches, officials and parents

**DOI:** 10.1186/s13643-022-01932-5

**Published:** 2022-05-19

**Authors:** Gavin Breslin, Stephen Shannon, Michael Cummings, Gerard Leavey

**Affiliations:** 1 Bamford Centre for Mental Health and Wellbeing, School of Psychology, Ulster University, Cromore Road, Coleraine, BT52 1SA Northern Ireland; 2 Sport and Exercise Sciences Research Institute, Ulster University Jordanstown Campus, Newtownabbey, Northern Ireland; 3 Maastricht University, Minderbroedersberg 4-6, Maastricht, 6211 LK The Netherlands

**Keywords:** Mental health literacy, Sport, Resilience, Athletes, Interventions, Health promotion

## Abstract

**Background:**

Interventions designed to increase mental health awareness in sport have grown substantially in the last 5 years, meaning that those involved in policy, research and intervention implementation are not fully informed by the latest systematic evaluation of research, risking a disservice to healthcare consumers. Hence, our aim was to update a 2017 systematic review that determined the effect of sport-specific mental health awareness programmes to improve mental health knowledge and help-seeking among sports coaches, athletes and officials. We extended the review to incorporate parents as a source of help-seeking and report the validity of outcome measures and quality of research design that occurred since the original review.

**Methods:**

Sport-specific mental health awareness programmes adopting an experimental or quasi-experimental design were included for synthesis. Five electronic databases were searched: Psychinfo, Medline (OVID interface), Scopus, Cochrane and Cinahl. Each database was searched from its year of inception to June 2020. As all of the outcomes measured were derived from psychometric scales, we observed statistically significant quantitative effects on the basis of *p* < .05, and a small, medium or large effect size as *d* = .2, .5 or .8, respectively. Risk of bias was assessed using the Cochrane and QATSQ tools.

**Results:**

Twenty-eight articles were included from the 2048 retrieved, eighteen additional articles since the original review. Eighteen studies targeted athletes, five with coaches, one sport officials (i.e. referees), one ‘at-risk children’ and three with parents. One of the studies was a combination of athletes, coaches and parents. In terms of study outcomes, health referral efficacy was improved in seven studies; twelve studies reported an increase in knowledge about mental health disorders. Proportionally, higher quality research designs were evident, as three of ten studies within the previous review did not demonstrate a high risk of bias, whereas thirteen of the eighteen additional studies did not display a high risk of bias. However, only one study included a behaviour change model in both the programme design and evaluation.

**Conclusions:**

Our updated systematic review provides evidence of the benefits of mental health awareness interventions in sport; these benefits are mainly for athletes and show improvements in the methodological design of recent studies compared to the first review. There was also evidence of the extension of programme delivery to parents. In conclusion, researchers, practitioners and policy makers should consider methodological guidance and the application of theory when developing and evaluating complex interventions.

**Systematic review registration:**

PROSPERO CRD42016040178

**Supplementary Information:**

The online version contains supplementary material available at 10.1186/s13643-022-01932-5.

## Background

Athletes have traditionally been portrayed as individuals who possess an abundance of human strength and mental toughness [1]. The reality is, however, that athletes struggle with mental health needs in a manner similar to the general population [2]. In some cases, higher prevalence rates of mental disorders are evident when athletes are going through periods of transition or adversity through injury, deselection, burnout, or prolonged competetion periods spent away from family and friends. Sport coaches are often viewed as a conduit to, or gatekeepers of, athlete mental health promotion [3]. However, coaches show similar mental health disorder prevalence rates as athletes [4] and, indeed, report the existence of unique  stressors mostly related to an imbalance between the coach’s ability to self-manage their mental health and meet the demands of the role [5]. Moreover, sports officials (e.g. referees, judges) face substantial adversity and harmful stressors which can include verbal abuse and aggression from athletes, fans and the media, that few feel equipped to deal with [4]. Therefore, mental health awareness training and the provision of suitable psychological and psychiatric support services are required for athletes, coaches and officials [6, 7].

In 2017, we published a systematic review of mental health awareness interventions delivered to sport clubs [8]. Ten interventions programmes were included that aimed to increase mental health literacy and support athletes, coaches and officials experiencing a mental health problem. While some support was found for the effectiveness of programmes in enhancing mental health awareness and help-seeking, few showed rigorous methodological quality, and most suffered a high risk of bias. None of the studies followed standards for reporting trials or referred to the Medical Research Council process evaluation framework [9]. Furthermore, few studies were underpinned and/or tested with a psychological theory of behaviour change and health. Michie et al. [10] have presented strong evidence that the integration of theory provides a clearer understanding of the causal assumptions underpinning intervention outcomes and provides a systematic evaluation framework to understand how and why interventions are effective in practice. Hence, our recommendation was for programme designers to give due consideration to the integration of behaviour change theory in the development and analysis of programmes. Moreover, we concluded that longitudinal studies are required with larger sample sizes of males and females, wherein randomisation to groups is blinded, and outcomes are measured with validated measurement tools [8].

Since the initial search, there has been a proliferation of research in the area of mental health and sport, evident in systematic reviews [2], peer-reviewed journal special issues (Journal of Physical Education Review, 2020) textbooks [7, 11, 12] and mental health–themed conferences (European Congress in Sport Science, 2017, British Psychological Society, Division of Sport and Exercise Psychology, 2018, 2020). Some Government and leading sport associations have developed mental health and wellbeing action plans or consensus statements to safeguard athlete mental health [2, 13, 14]. More recently, an emphasis was placed on supporting *all* those participating in sport, through a call to action to move beyond only supporting the elite athlete [15]. An international consensus statement was also published describing that mental health awareness programmes should be available for all involved in sport (i.e. athletes, coaches, officials, parents), that programme content should be theory-based and evidence-informed and include robust evaluation [6].

With respect to such developments in the mental health in sport field of study, Garner and colleagues [16] have outlined that newly identified studies can potentially change conclusions and recommendations of a previous review. Given systematic reviews are central to healthcare science, and inform practitioners, intervention and policy development, those involved in design and implementation are not fully informed by the latest research. Furthermore, outdated reviews do not capture novel theoretical developments and/or topical issues where further research may be imminently needed. Given the upsurge in research in mental health awareness raising, our first aim was to update and extend our original systematic review conducted determining the effect of mental health awareness programmes to improve mental health knowledge and help-seeking among sports coaches, athletes and officials [8]. Using Garner and colleagues [16] consensus and checklist for updating systematic reviews, we replicated and extended the original review to include athlete parents. Interventions targeted at improving the mental health awareness of parents are important as they target three key aspects of mental health literacy that will allow the parent to provide the optimal support for their children who are participating in sport [17]. Namely, enhanced symptom recognition can allow the parent to recognise ‘warning signs’ of key mental health disorders to allow them to provide support for their child should they become affected. The ability for a parent to effectively help their child with a mental health issue has been shown to be greatly influenced by their attitudes toward mental health. Efforts to reduce any stigma among parents of athletes could be greatly beneficial for reducing barriers to help-seeking in their children. Finally, knowing how and where to seek appropriate information on mental health disorders and treatment options [17]. Not knowing what to do, or where to turn for help has been identified by parents as the most common barrier to facilitating help-seeking behaviour and highlights how improved mental health literacy knowledge and signposting could be of particular importance [18]. In reviews to date, the role of the parent has not been included. The second aim was to review newly retrieved study quality and report on the validity of measures that were used to determine the effectiveness of programmes. A description of intervention programmes delivered are provided and recommendations for those in the process of designing and evaluating mental health programmes for athletes, coaches, officials and parents are proposed.

## Methods

### Protocol

All methods of data analysis and reporting followed the Preferred Reporting Items for Systematic Reviews and Meta-Analyses (PRISMA) guidelines [19]. Amendments to the original PROSPERO protocol (International database of prospectively registered systematic reviews in health and social care) were included in December 2021 and can be accessed (Registration number: CRD42016040178). A PRISMA checklist is provided as a supplementary file.

### Eligibility criteria

#### Types of studies

Randomised or clustered randomised controlled trials and quasi-experimental studies that did not use a pre-specified randomisation processes when selecting the treatment and comparator condition [20] were included. Studies comparing the treatment with a comparison group, more than one intervention group or within subjects across time (i.e. pre-post testing) were included. Studies were required to have been published in the English language. The decision was taken to restrict our inclusion criteria to only peer-reviewed literature as grey literature (e.g. dissertations, reports, policy documents) is heterogeneous, and little methodological guidance exists for the systematic retrieval, analyses and reproducibility of such work [21].

#### Types of participants

Participants were children, adolescents or adults who are considered an athlete, leader, coach, parent, official or member (e.g. service provider) within a professional, semi-professional or amateur community sporting club or organisation.

#### Types of interventions

Mental health interventions that took a general approach to improving awareness of mental health (e.g. help-seeking, knowledge of disorders, literacy), or interventions tailored to improve mental well-being (e.g. positive affect, life satisfaction), or reduce symptoms of mental ill-being (e.g. depression, anxiety) were included. While eating disorders are a relevant topic for mental health awareness programmes, we decided to exclude these studies because several recent systematic reviews focus on the specific nature and implementation of eating disorder prevention initiatives for athletes [22, 23]. The mode of delivery was individual, group or web-based. To be eligible for inclusion, interventions had to take place within a sport setting (i.e. sport club, sport environment), or be focused for athletes, coaches, officials, parents or service providers. As many definitions of sport exist, we applied Rejeski and Brawley’s [24] definition for consistency: structured physical activity that is competitive, rule-governed and characterised by strategy, prowess and chance. Exclusion criteria applied to interventions that were considered being outside the domain of sport (e.g. physical activity, exercise, leisure, art, dance and music).

#### Types of outcome measures

Studies needed to include at least one outcome measure which we categorised as related to mental health attitudes (e.g. stigma, prejudice), knowledge of mental health (e.g. disorder/symptom recognition), or behaviour regarding mental health (e.g. intended/ actual help-seeking for oneself or others); mental health–promoting competencies/skills (e.g. mindfulness, coping), or specific mental health (e.g. anxiety, depressive symptoms) and/or well-being (i.e. subjective/psychological well-being domains, life satisfaction) outcomes. Only quantitative studies were included as it would be difficult to assume a level of generalisability between quantitative and qualitative outcomes. Furthermore, a qualitative review could be reported as a separate article.

### Information sources and search strategy

We used electronic databases and also manually checked reference lists of articles. Five electronic databases were searched: PsychInfo, Medline (OVID interface), Scopus, Cochrane and CINAHL. Each database was searched (see Table 1) from its year of inception to July 2020. Search terms used keywords, truncation and MeSH terms as appropriate for each database’s indexing reference [25]. The search was stratified into four categories: sport, participants, setting and method of treatment. Search terms were the same as the original review and chosen based on previous research, theory and practice. The first category used sport as a single term as sport is central to the objective of the review. As with previous systematic reviews in sport [26], the second category used descriptors associated with participation or membership within sport. The third category depicted broadly cited sport settings in sport development literature [27] and also included internet-based terms to account for recent developments of online mental health interventions [28]. Lastly, search terms in the fourth category were applicable terms to constructs associated with mental health and well-being [29], mental health knowledge [30] and competence strategies appropriate for mental health interventions [31]. A full electronic search of the Psycinfo search is uploaded as a supplementary file. Reference lists of included articles were also searched.

**Table 1:** Search terms used in Psycinfo search reflecting keywords, mesh terms and suffixes

Category	Key terms
Sport	Sport$
Participants	Leader$ or athlete$ or teacher$ or instructor$ or player$ or member$ or participant$ or coach$ or official$ or parent$
Setting	Sport adj3 (organi#ation$ or club$ or governing bod$ or cent$ or school$ or setting$ or internet or online or website$ or web site$ or web based)
Method of treatment	mental$ adj3 (health or wellbeing or well being or well-being or wellness or ill$) or anxiety or depress$
Limiters	English language and peer reviewed

*$* Search singular or plural, *adj3* Adjacent, # Replaces 1 character

### Study selection and data collection process

**Fig. 1:**
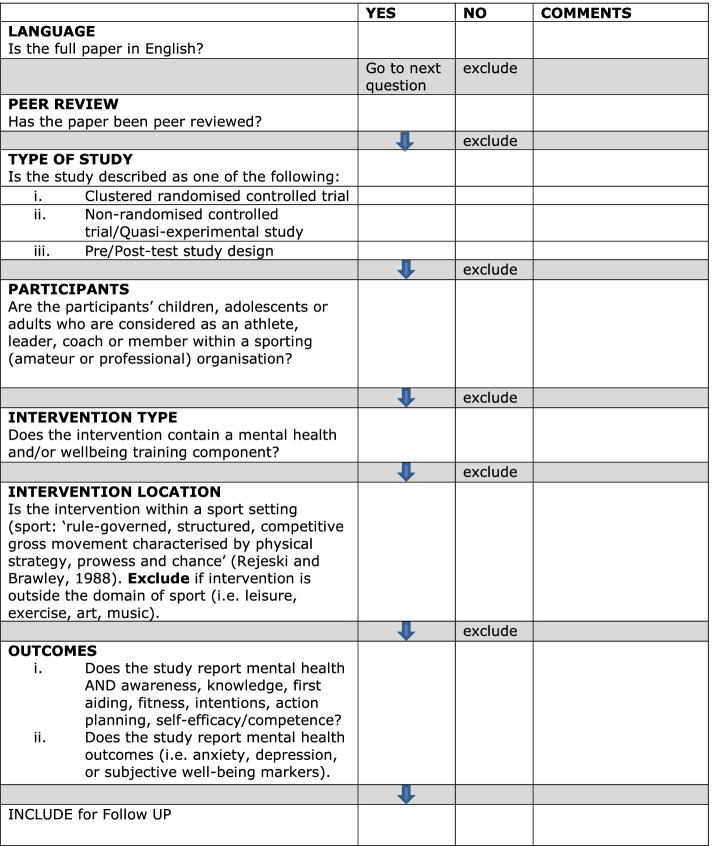
Screening tool for independent author screening

Study selection was completed in three phases. First, database searches were exported to Refworks software into a master folder. All titles and abstracts were screened by one researcher. Duplicates were removed and all abstracts were exported to a subfolder (i.e. included for follow-up or excluded). All relevant abstracts were printed and screened by a second and third researcher to assess their eligibility for full-text printing and screening. Second, to ensure inter-rater reliability two researchers independently screened 10% of all excluded titles and abstracts. Although a high level of agreement (>95%) was reached, potentially relevant abstracts were highlighted and subsequently screened by two authors using the inclusion criteria. They were found to be irrelevant and were excluded. Third, full-text eligibility assessment was performed independently in an un-blinded standardised manner by three researchers (GB, SS, MC) using the screening tool (see Fig. 1). The remaining included articles were divided between three researchers and all pre-defined data (see below) was extracted by one researcher and cross-verified by a second and third for the synthesis of results.

### Data items, summary measures, synthesis and analysis of results

Detailed descriptive information from each intervention including the author(s) and year of study; study design features (e.g. data collection points, inclusion of a control group or not); sample characteristics including age and gender; mode of delivery and mental health descriptor (i.e. increase knowledge, improve attitudes or reduce depressive symptoms) were extracted. For assessing the effect of the interventions we obtained the name of the outcome measure(s), reported value(s) for intervention effectiveness (e.g. *p* value, effect size) and, based on prior research [32], provided a narrative commentary on study design methods that may influence the generalisability of study effects. As all of the outcomes measured were derived from psychometric scales, we observed statistically significant quantitative effects on the basis of *p* < .05 [33], and a small, medium or large effect size as *d* = .2, .5 or .8, respectively [34]. We reported the effects of each study in Table 3. For combining and reporting the results, we inspected each study’s outcomes and categorised them in accordance with the following key mental health constructs [35]: stigma, mental health knowledge, referral efficacy/confidence, help-seeking intentions and behaviour, well-being and additional outcomes.

### Risk of bias within and across studies

For profiling, the study quality and risk of bias the principles of the Cochrane Collaboration for assessing methodological quality in systematic reviews were adopted [20]. As included studies were either categorised as randomised or non-randomised designs, each study’s design was matched with an applicable assessment of bias tool. For randomised controlled trials we used the Cochrane Collaboration’s tool for assessing risk of bias [36]. The tool includes six domains of bias such as selection, detection and reporting bias. Each domain is coded as high, low or unclear for the relative risk of bias and an overall judgement is accumulated. For non-randomised studies, we used the ‘Quality Assessment Tool for Quantitative Studies’ (QATSQ) [37] that is recommended for use in systematic reviews [38]. The QATSQ tool is scored based on six domains of bias including selection bias, confounding bias and withdrawals and dropouts. Based on the pre-defined bias criteria, the domains were scored as either weak (3), moderate (2) or strong (1). Studies with no weak ratings and at least four strong were considered strong, while studies with fewer than four strong ratings and one weak rating were considered moderate, and studies with two or more weak ratings were considered weak [37]. Based on the Cochrane Collaboration’s recommendations [36] we reported on the risk of bias across studies by summarising the cumulative bias for each outcome in the Cochrane and QATSQ tools. To facilitate reporting of bias across the studies, additional rows and columns were added to the tools.

Outcome measures were also assessed for validity as they can influence the generalisability of study findings [33]. The study adapted criteria used in a recent systematic review of mental health interventions [28] and also used in the original review [8]. Scales were considered acceptable if they met one or more of the following: a Cronbach’s alpha value of above .7; reporting of acceptable goodness of fit indices using confirmatory factor analysis [39], test-retest, construct or concurrent validity assessments; and/or the authors referenced a previous study that validated the scales through the above methods.

## Results

**Fig. 2:**
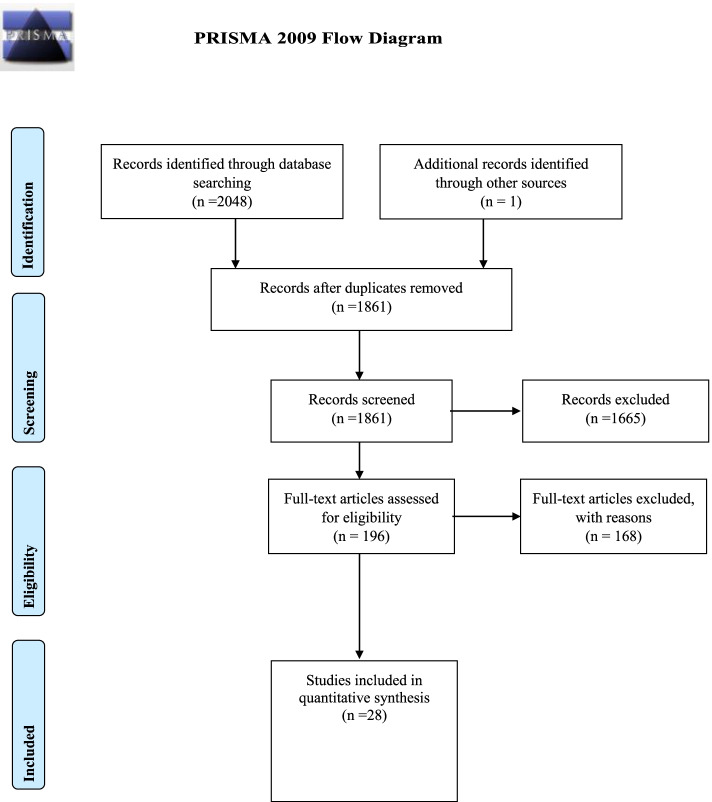
PRISMA 2009 flow diagram

A total of 2048 titles and abstracts were reviewed (See Fig. 2). One further article was identified through a co-author’s knowledge of the area of research. After removal of duplicates (*n* = 188), 1861 titles and abstracts remained. Of these, 1665 were identified as not meeting the eligibility criteria and were excluded. A total of 196 articles were identified as eligible and therefore underwent a further detailed screening, 33 articles met the criteria for full-text screening by two researchers. Of the 33 articles, researchers agreed upon six articles to be excluded because they did not meet the inclusion criteria on at least one aspect. Two of the articles were related to a study that was already included in the quantitative synthesis [40] that included a trial registration and a book chapter providing a description of the aforesaid intervention delivered within the study. Three articles were removed on the basis that they were tailored toward sport performance–related outcomes (psychological skills training) rather than mental health awareness and therefore fell outside of the scope of the review [41, 42]. One study was deemed ineligible as the participants were not considered to be involved in sport [43]. The remaining 27 studies [8, 18, 44–58] achieved 100% researcher agreement for their inclusion for review, 10 of these articles were those included in the original systematic review [8] and underwent quantitative synthesis [59, 60, 62–69]. A further 15 references were identified upon hand-searching the reference lists of the 27 included articles, wherein one further article [70] met the inclusion criteria. However, 14 were excluded from any further data synthesis as they fell under the category of chapters in books, cross-sectional surveys or contained qualitative findings (see Fig. 2).

### Study characteristics

Study characteristics are detailed in Table 3. Across the 28 studies, 4657 participants took part, consisting of 1234 males and 1113 females. Three studies did not detail subject gender, including 1004 [57], 995 [68] and 311 [65] participants respectively. The interventions were delivered to a variety of sports participants. Half of the studies within the original systematic review targeted coaches and service providers (*n* = 5), others focused on elite and non-elite athletes (*n* = 3), one was aimed at officials (*n* = 1) and another ‘at-risk’ children (*n* = 1). The newly synthesised articles shifted toward studies that specifically targeted athletes (*n* = 16), collectively making athletes the most researched subgroup (*n* = 19). Two studies focused on parents of sport participants exclusively (*n* = 2). One study detailed a multicomponent intervention that included adolescent athletes, parents of athletes and coaches (*n* = 1). No further studies were found that investigated interventions in officials. Studies deployed a variety of designs including intervention pre-post testing (*n* = 10), randomised control trial (*n* = 8), controlled trial (*n* = 7), quasi-experimental study (*n* = 2) and a descriptive case trial (*n* = 1). The mode of delivery for the majority of studies was via group setting (*n* = 23); however, many of these interventions also included a blend of online and home-based elements. An individual counselling format (*n* = 2), web-based (*n* = 3) and home (*n* = 1) settings made up the remainder.

### Study results

The name of the author(s) who conducted the study, the year, the design, study duration, sample characteristics, mental health descriptor employed, mode of delivery, mental health outcome measure(s), main findings and general comments regarding each study are summarised in Table 2. Studies selected for inclusion were published between November 1999 and May 2020.

**Table 2:** Descriptive information for included studies

Authors (year of study)	Study design; duration	Sample characteristics	Mental health descriptor; mode of delivery
Ajilchi et al. (2019) [45]	Randomised controlled trial; 6 weeks	Amateur basketball players (*n* = 30; 30 males, age = 22)	Emotional intelligence; mindfulness programme consisting of weekly 90-min group sessions and home practice delivered under supervision of experienced psychologist
Bapat et al. (2009) [59]	Pre-post design; 3 weeks	Sport club leaders (*n* = 40; age = 38.62; 16 males, 24 females)	Mental health literacy through mental health first aid training; 8-h training programme delivered over 3 sessions using a range of presentations, tasks and homework
Breslin et al. (2017) [8, 60]	Controlled trial; 1 day (3-h session)	Sport coaches (*n* = 244; 126 males, 118 females)	Mental health awareness programme involving videos and discussions with athletes who have experienced depression; 3-h programme delivered in one session by a public health agency provider
Breslin et al. (2018) [61]	Pre-Post design; 1 day (75-min session)	Student athletes (*n* = 100;59 males, 41 females, age = 20.78)	Multicomponent mental health awareness program; 75-min experiential and skill-enhancing programme delivered by experienced mental health and well-being tutors.
Chow et al. (2020) [46]	Pre-Post design; 4 weeks	Student athletes (*n* = 33; age = 19.2, 13 males, 20 females)	Mental health literacy and stigma reduction; 4 60-min sessions delivered by experienced mental health researchers incorporating psychoeducation, group discussion and video learning
Donohue et al. (2015) [62]	Single subject pre-post and follow up design; 4 months	Athletes with previous history of substance abuse or dependence (*n* = 7; age = 20; 4 males, 3 females)	Modifying behavioural and cognitive skills to overcome substance abuse; 12 individual meetings on a range of topics
Donohue et al. (2018) [47]	Randomised controlled trial; 8 months	Collegiate athletes (*n* = 74;38 males,36 females, age = 20.64)	Mental health symptom severity; 2 intervention arms, one consisting of 12 60–90-min sport and life performance optimization meetings, the other was consistent with customary university mental health services, consisting of 12 50 minute office-based outpatient sessions
Dowell et al. (2020) [48]	Pre-Post design;5 months	Youth rugby league players (*n* = 74;74 males, age = 13.23)	Mental health symptom severity; connected participants and parents to multi-component intervention including online resources, group-based workshop (4 × 30 min) and tailored individual follow-up
Dubuc-Charbonneau and Durand-Bush (2015) [49]	Pre-Post design;1 season (approximately 6 months)	Student athletes (*n* = 8;4 males, 4 females, age = 21.5)	Mental well-being; person-centred, self-regulation intervention, 40–60-min sessions led by experienced researcher
Fogaca (2019) [50]	Quasi-experimental design;5 weeks	Student athletes (*n* = 88; age = 19.8, 45 females, 43 males)	Mental health outcomes; teaching coping skills and increasing social support within team delivered by author
Glass et al. (2019) [51]	Randomised controlled trial;6 weeks	Student athletes (*n* = 52; age = 19.32, 44 females, 8 males)	Mental well-being; 6 90 minute educational, discussion-based sessions with meditation exercises progressively introduced, co-facilitated by 2 clinical psychologists
Gross et al. (2018) [44]	Randomised controlled trial;7 weeks	Collegiate athletes (*n* = 18;18 females)	Psychological well-being outcomes; 2 intervention arms, both involved 7 60 minute sessions, one focused on psychoeducation and mindfulness the other, mental skills training
Gulliver et al. (2012) [63]	Randomised control trial; 5 weeks	Elite athletes (*n* = 59; age = 25.5; 16 males, 43 females)	Mental health literacy; participants were allocated to one of a series of online psycho-educational programmes
Hurley et al. (2018) [18]	Controlled trial;1 month	Parents of adolescent sports club members (*n* = 66; age = 44.86, 49 females, 17 males)	Mental health literacy; 65 minute mental health literacy intervention workshop delivered through community sports clubs along with informative pamphlet and online resources
Hurley et al. (2020) [52]	Controlled trial;1 month	Parents of adolescent sports club members (*n* = 540;age = 47.42,321 females, 219 males)	Mental health literacy; 50–75-min mental health literacy intervention workshop delivered through community sports clubs employing a community based participatory approach along with informative pamphlet and online resources
Laureano et al. (2014) [53]	Quasi-experimental design;2 weeks	University student rugby players (*n* = 76;age = 20.69, 76 males)	Coping self-efficacy and psychological well-being; experiential learning programme consisting of 6 1-hr group sessions, participants received psychoeducational workbook
Longshore et al. (2015) [64]	Controlled trial; 6 weeks	College coaches (*n* = 20; age = 34.5; 8 males, 12 females)	Mindfulness training programme to develop emotional awareness and reduce stress; an initial 1.5 h group session followed by a 6 week home program
Liddle et al. (2019) [40]	Cluster-randomised controlled trial; 6 weeks	Adolescent sport participants (*n* = 102; age = 14.3, 102 males)	Mental health literacy; 45-min workshop in a community sports club via powerpoint, facilitated discussions and role-play
Mohammed et al. (2018) [54]	Pre-Post design; 8 weeks	Injured student athletes (*n* = 20; age range = 21–36, 14 males, 6 females)	Mental well-being and mindful awareness; weekly formal and cd-guided informal meditation practise
Pierce et al. (2010) [65]	Pre-post design (club leaders); controlled trial (football players); 3 weeks	Club leaders (*n* = 36; age = 45); and football players (*n* = 275; age = 21)	Mental health literacy; 12-h psycho-educational group sessions for leaders; information sessions were conducted with players alongside informal information
Sebbens et al. (2016) [66]	Controlled trial; 1 day (4 h)	Coaches, trainers, support staff and service provides (*n* = 166; age = 37.8; 83 males, 83 females)	Mental health knowledge and confidence program; 4-h applied workshop involving case studies, role-playing and videos
Sekizaki et al. (2019) [55]	Randomised controlled trial; 4 weeks	High school athletes (*n* = 80;age = 15.75, 80 males)	Mental well-being and self-efficacy; 180-min group education in a school setting regarding cognitive behaviour therapy and online homework using iCBT
Shannon et al. (2019) [56]	Controlled trial; 2 weeks	Student athletes (*n* = 238; age = 20.47, 137 males, 101 females)	Mental well-being and mindful awareness; 90-min intervention workshop through a needs-supportive environment delivered by a psychiatrist and counsellor followed by mindfulness programme via mobile app
Slack et al. (2015) [67]	Single subject pre-post design; 1 season (approximately 6 months)	Referees (*n* = 3; age = 28.67; 3 males)	Mental toughness education and training program; six monthly workshops involving four individual-based and two group-based sessions consisting of role-playing and cognitive behavioural therapy techniques
Tester et al. (1999) [68]	Pre-post design; 2 school years	‘At risk’ schoolchildren enrolled in a sports programme (*n* = 991)	Preparation for life skills (i.e. pro social behaviours, stress management) were taught by sporting mentors through a basketball programme in and outside classroom settings over the course of 2 years
Van Raalte et al. (2015) [69]	Randomised controlled trial; 1 day (online session lasted at least 10 min)	Student athletes (*n* = 153; age = 19.63; 46 males, 103 females)	Mental health literacy; web-based programme using exercises and interactive material
Vella et al. (2020) [57]	Controlled trial; 8 weeks	Adolescent male sport participants, parents of participants and coaches (*n* = 1004)	Mental health literacy; multi-component sports-based programme to promote early intervention, help-seeking and resilience
Vidic et al. (2018) [58]	Pre-post design; 9 weeks	Collegiate male soccer athletes (*n* = 18; age = 19.56, 18 males)	Stress; 6 1 hour mindfulness meditation-based sessions led by experienced practitioner

Twenty-one studies utilised a control group [8, 18, 40, 44, 45, 47, 49–57, 60, 63, 64, 66, 69, 70] of which nine implemented randomisation methods [40, 44, 45, 47, 51, 54, 55, 63, 69]. Sample sizes significantly varied, from the extremely large (*n* = 1004) [57] to small (*n* = 3) [67]. Nine studies collected outcome measures pre- and post-intervention and obtained measurements at a further follow-up point in time, i.e. [18, 40, 44, 46, 47, 51, 62, 63, 66]. Three of the studies from the original review did not report a statistical test of significance [62, 67, 68], all further studies included values for statistical significance testing. A total of 13 studies ( [40, 49, 55, 57–60, 62, 65–68, 70] did not report effect sizes. Collectively, findings across the studies support the importance and efficacy of introducing knowledge-based mental health programmes in sport settings. Of the studies that included a follow-up, the majority maintained some of their effects (*n* = 8), while one did not (*n* = 1) [66], showcasing the potential long-term efficacy of such interventions. The impact of the interventions on the multitude of targeted outcomes is detailed below.

### Effects on stigma

One intervention elicited a reduction in stigma around anxiety, yet stigma for depression remained unchanged [63]. Stigma for depression was significantly reduced in another study [59]. Two studies reported on stigma for socialising with individuals with a mental health disorder, one study showed a significant reduction [60] while the other did not [57]. However, those who completed the entirety of the Vella et al. programme reported a decrease in attitudes to stigma. The same research team in the Liddle et al. study examined stigmatising attitudes and showed that they were significantly reduced post-intervention, with changes sustained at follow-up [40]. In the Chow et al. [46] study, improvement in self-stigma of seeking help was improved and was sustained at follow-up, conversely, personal, public and implicit stigma did not differ significantly between pre- and post-intervention.

### Effects on mental health knowledge

Eleven [8, 18, 40, 46, 57, 59, 60, 63, 65, 66, 69] of the twelve studies reporting on mental health knowledge, showcased a significant rise in aspects of mental health knowledge, attitudes toward mental health, disorder-specific recognition and mental health referral knowledge. The study by Hurley and colleagues [52] displayed no significant improvements for the intervention group in comparison to control in mental health literacy outcomes. Six studies deployed the Anxiety Literacy Questionnaire (A-LIT) [63] and Depression Literacy Questionnaire (D-LIT) [71]. There was substantial heterogeneity in the further six studies’ assessment and conceptualisation of mental health knowledge (see Table 3).

**Table 3:** Study outcome measures, main findings and comments on study

Authors (year of study)	Mental health outcome measure(s)	Main findings	Comments
Ajilchi et al. (2019) [45]	SEIS	Significant improvement for intervention group in comparison to control for emotional intelligence following MSPE (*p* < 0.0005)	Small sample (*n* = 30); not a diverse sample; no blinding of participants or research personnel; no follow-up data
Bapat et al. (2009) [59]	SQ; KQ; ?V	Significant reduction in levels of stigma (*p* < 0.001); increase in knowledge about mental disorders (*p* < 0 .01); increased confidence to help someone with mental disorder (*p* < 0.001)	Small sample size (*n* = 40); no control group; no effect sizes reported; no follow-up data
Breslin et al. (2017) [8, 60]	RIBS; MAKS; ?3	Significant improvement for intervention group in comparison to control on mental health knowledge, confidence in ability to help someone, and intention to offer help to individuals with a mental health problem (all findings *p* < 0.001)	No randomisation method; no follow-up data; no effect sizes reported; intended behaviour was reported rather than actual behaviour
Breslin et al. (2018)	RIBS; MAKS; SWEMWBS; BRS	Significant improvement for intervention group in comparison to control on mental health knowledge (*p* < 0.001) and intention to offer help to someone with mental health disorder (*p* < 0.01). No significant improvement in well-being or resilience post-intervention.	No randomisation method; no follow-up data; intended behaviour rather than actual behaviour; high attendance due to scheduled class; one session insufficient to influence resilience
Chowba et al. (2020) [46]	MHL; SSSH; PSTIG; PBS; IS;ATSPPH; ISC	Significant promotion of mental health literacy, intentions to seek counselling (*p* < 0.0001), self-stigma and attitudes toward seeking help (*p* < 0.01) from pre-post intervention with sustained improvements at 1-month follow up. Particularly large effect on MHL. Personal, public and implicit stigma did not differ significantly.	No control group; small sample size(*n* = 33); magnitude of effect sizes encouraging
Donohue et al. (2015) [62]	SCL-90-R;BDI;SARI;TLFB;RAB	Psychiatric functioning mean scores improved from baseline to post. Improved scores remained stable at 1- and 3-month follow-up; depressive mean scores decreased from baseline to post-intervention and remained stable at follow-up. Improvements were shown for all relationship domains.	Small sample size (*n* = 7); no values provided for study effects (i.e. p value or effect); no control group
Donohue et al. (2018) [47]	SCL-90;BDI;SIC-LOS;SARI;TFLB;RAB;OHSO	Participants in The Optimum Performance Program in Sports (TOPPS) reported greater improvements in overall mental health, mood and life outside sport significant up to 8 months follow-up than those with Psychological services as usual (SAU). Greater improvements in happiness with significant others and their contributions to sport from baseline to 4 months (*p* < 0.05). TOPPS more efficacious in reducing substance abuse yet neither programme reduced risky sexual behaviour.	Most outcome measures do not assess wellness beyond absence of pathology; no impact on risky sexual behaviour- complement further programmes with evidence based prevention
Dowell et al. (2020) [48]	RCADSAS;RCADDS;SDQCPS;AGS;YLOT;GC-6;SDQPBS;NES	Participants showed significant reduction in anxiety from pre-post intervention (*p* < 0.05) and marginally significant reduction in depressive symptoms with no difference between urban and rural areas. No significant effects on anger, optimism or gratitude. Significant improvements in secondary outcomes of prosocial behaviour and managing negative emotions. Grit increased significantly within urban population but not rural.	No control group; Preliminary findings as a result
Dubuc-Charbonneau and Durand-Bush (2015) [49]	PSS;WEMWBS;SSRQ	Significant reduction in stress, increase in well-being and capacity to self-regulate (*p* < 0.05).	Small sample size (*n* = 8); narrow range of eligible sports; no long-term follow-up
Fogaca (2019) [50]	ACSI-28;BAI;BDI;WHOQOL	Significant increase in coping ability and reduction in anxiety (*p* < 0.05). Small effect sizes (*n*^2^*p* < 0.3). Neither depression nor quality of life showed significant change.	No randomisation method; not a diverse sample
Glass et al. (2019) [51]	DASS-21;SWLS;FFMQ;AAQ-II	Participants in control group showed significant increases in depressive symptoms whereas there was a slight but non-significant decrease in the intervention group. Significant increase in life satisfaction (*p* < 0.05) and observing aspect of mindfulness (*p* < 0.01). No significant change in acceptance and action.	Relatively small sample size (*n* = 52); high attrition rate; limited engagement as participants with unfamiliar people
Gross et al. (2018) [44]	CCAPS-62;AAQ-II;DERS;MAAS	MAC group had significant effect on substance use, distress, anxiety and hostility compared to PST (*p* < 0.05). MAC had a significant impact on emotional regulation and acceptance (*p* < 0.05). PST had a significant impact on mindfulness compared to MAC (*p* < 0.05).	Small sample size (*n* = 18); Low sample diversity; No no-treatment control group; PST not developed for mental health purpose
Gulliver et al. (2012) [63]	ATSPPH-SF;GHSQ;AHSQ;D-Lit;A-Lit;DSS;GASS	No significant interaction effect for help-seeking attitudes, intentions or behaviour from baseline to follow-up. However, significant positive interaction effects were observed for depression (*p* < 0.05) and anxiety literacy (*p* < 0.01), and anxiety stigma (*p* < 0.05) from baseline to follow up relative to control group	Effect sizes for the significant positive interaction effects differed for treatment condition (literacy condition, feedback condition and help-seeking) in comparison to control, ranging from small to medium to large. Caution is advised when interpreting findings as the sample size was small
Hurley et al. (2018) [18]	A-LIT;D-LIT;K6; PCPH	Participants in the intervention group significantly improved their depression and anxiety literacy; knowledge of help-seeking options and confidence to provide support for someone experiencing a mental health disorder to a greater extent than those in a matched control group. These improvements were maintained at 1 month follow-up (all findings *p* < 0.05). Intervention did not significantly reduce distress or improve attitudes to facilitate mental health promotion and help-seeking.	Attitudes rather than actual behaviour reported; no randomisation method
Hurley et al. (2020) [52]	A-LIT; D-LIT; MHLS; GHSQ; PCPH; PSSN; K6	Parental depression and anxiety literacy, intentions to seek help for adolescent and attitudes toward mental health and help-seeking did not significantly improve in intervention compared to control rather improvements were observed in both at follow-up. Intervention group displayed improved knowledge and confidence to assist (*p* < 0.001) and were more likely to seek formal help, felt increased social support and reduced distress (*p* < 0.05).	Longer term follow up not feasible; low retention of participants; no randomisation method
Laureano et al. (2014) [53]	CSE; FORQ; AFM-2	Intervention group showed that sum of coping self-efficacy, fortitude and overall well-being improved significantly (*p* < 0.01). After adjustment for pre-test difference fortitude differences were no longer significant and overall happiness less significant (*p* < 0.1).	Demographic information not gathered; no longer term follow up; no randomisation method
Liddle et al. (2019) [40]	PCHB; OMHE; IPH; D-LIT; A-LIT; GHSQ; CPH; MHLS; K6	Intervention improved depression and anxiety literacy post-intervention with significant anxiety effects sustained over 1 month (*p* < 0.01), improving stigmatising attitudes also were retained (*p* < 0.001). Intentions to provide help improved but were not sustained. Help-seeking intentions did not improve. Both groups were more likely to seek support from informal sources.	Control group not matched in age; no longer term follow-up
Longshore and Sachs (2015) [64]	MAAS;TMS;STAI;PANAS;BRUMS	No significant interaction effect reported for anxiety, mindfulness awareness or experience, or moods. A significant interaction effect was reported for a reduction in negative affect (*p* < 0.05, ES = 0.21)	Small sample size (*n* = 20). Despite largely nonsignificant results, mean scores showed positive trends, and effect sizes were generally small to moderate. Interviews with participants showed positive changes in coaches’ personal life and mindfulness
Mohammed et al. (2018) [54]	MAAS; DASS; POMS	Mindful awareness was higher immediately post session in intervention than control and further increased after 8 weeks in the intervention group (*p* < 0.001). Decrease in depression, tension, fatigue and confusion mood states after sessions and time (*p* < 0.005). There was a notable decrease in anxiety and stress across sessions, however only stress was maintained over time (*p* < 0.05).	Small sample size (*n* = 20); type of injury should be taken into consideration
Pierce et al. (2010) [65]	?1;?2	Leaders: Significant positive change in recognition of mental illness (*p* < 0.001), confidence that antidepressant medication can be helpful (*p* < 0.01) and confidence in helping someone with mental health problem (*p* < 0.001). Players: no significant changes	Leaders: Small sample size (*n* = 36), no control group. Players: Unclear information on their attendance and involvement in the intervention. No effect sizes reported
Sebbens et al. (2016) [66]	D-Lit; A-Lit; ?3	A significant interaction effect was recorded for the intervention group in comparison to control on depression and anxiety literacy and confidence to help at time 2 (2 weeks post-intervention) (*p* < 0.001) but not at time 3 (4 weeks post-intervention)	No randomisation method; no effect sizes reported; intended behaviour was reported rather than actual behaviour
Sekizaki et al. (2019) [55]	K6; GHQ-12; GSES	Increase in K6 scores for depression in control group but remained the same in intervention. Statistically significant reaction observed for group x time for distress (*p* < 0.01) but there was no significant pre- and post-intervention changes. No significant interactions occurred in GHQ-12 or self-efficacy	Non-blinded; short intervention period
Slack et al. (2015) [67]	SGMT; RSMT	Positive mean score changes were recorded for all three referees’ general and referee-specific mental toughness scores in the intervention phase in comparison to baseline	No values provided for study effects (i.e. p value); no control group; qualitative data strengthened the evaluation of program; referees’ performance increased
Shannon et al. (2019) [56]	MAAS; PCS; PSS; WEMWBS	Mindful awareness was not directly enhanced by the intervention in Model 1 (mindfulness M1) resulting in no indirect effects on competence, stress and well-being. In Model 2 (competence M1), the intervention was directly related to positive changes in competence, resulting in indirect effects on mindful awareness, stress and well-being (all findings *p* < 0.05). Indirect effects for intervention group on stress through competence and mindful awareness and on well-being through competence, mindful awareness and stress in sequence (*p* < 0.05).	Key contribution was inclusion of SDT to test mechanisms of change; Effect sizes small; Lack of long-term follow-up; No randomisation method; Low adherence to full program
Tester, Watkins and Rouse (1999) [68]	SCQ	Overall mean improvement of 44% (6–11-year-olds) and 18% (12–16-year-olds) in post-test scores in comparison to baseline for self-concept	No values provided for study effects (i.e. p value, effect size); no control group
Van Raalte et al. (2015) [69]	MHRES;MHRK	Significant positive changes were observed for mental health referral efficacy (*p* < 0.001, ES = 0.1) and knowledge (*p* < 0.01, ES = 0.04) for the intervention group in comparison to control group	Intervention was tailored for the population. Qualitative data showed positive feedback for intervention acceptability
Vella et al. (2020) [57]	D-LIT; A-LIT; CDRS; MHLS; GHSQ; SDS; IB; MDSPSS; K6; MHC	Significant improvements in depression and anxiety literacy (*p* < 0.001), intentions to seek formal help (*p* < 0.01), confidence to seek information, resilience and wellbeing (*p* < 0.05). No significant group by time effects were found for stigma, intentions to seek informal help, implicit beliefs, perceived familial support or psychological distress.	High baseline scores limit effects through ceiling effect; longer term follow-up required; large sample size (*n* = 1004); no account for variations in implementation
Vidic et al. (2018) [58]	PSS	Study demonstrated decreases in overall mean perceived stress levels from pre-test to post-test but these findings were not statistically significant (*p* = 0.44)	Lack of control group; small sample size (*n* = 18); no randomisation method
Summary	Broad range of measures used to assess mental health outcomes	Significant findings for all mental health outcomes measured (*n* = 4); significant findings on at least one outcome measure (*n* = 22). Non-significant findings (*n* = 3). Actual behaviour change for help-seeking (*n* = 0)	Small sample size (*n* = 10), no control group (*n* = 8), randomisation (*n* = 9), follow-up measures included (*n* = 9)

*SEIS* Self-rated emotional intelligence, *SQ* Stigma questionnaire, *KQ* Knowledge questionnaire, *?V* No name given to confidence measure for vignette, *?3* No name given to measure with questions around mental health confidence to help, *RIBS* Reported and Intended Behaviour Scale, *MAKS* Mental Health Knowledge Scale, *SWEMWBS* Short Warwick Edinburgh Mental Well-being Scale, *BRS* Brief Resilience Scale, *MHL* Mental health literacy, *SSSH* Self-stigma of seeking help, *PSTIG* Personal stigma, *PBS* Public stigma, *IS* Implicit stigma, *ATSPPH* Attitudes toward seeking professional psychological help, *ISC* Intentions to seeking counselling, *SCL-90* Global Severity Index of Symptom Checklist 90, *BDI* Beck Depression Inventory, *SIC-LOS* Sport Interference Checklist Life Outside Sport, *SARI* Student athlete relationship index, *RAB* Sexual Risk Scale of Risk Assessment Battery, *OHSO* Overall happiness with significant others, *RCADSAS* Revised Children’s Anxiety and Depression Scale Anxiety Subscale, *RCADSSS* Revised Children’s Anxiety and Depression Scale Depression Subscale, *SDQCPS* Strength and Difficulties Questionnaire Conduct Problems Subscale, *AGS* Academic Grit Scale, *YLOT* Youth Life Orientation Test Optimism Subscale, *GC-6* Gratitude, *SDQPBS* Strength and Difficulties Questionnaire Prosocial behaviour Subscale, *NES* Multidimensional Self-Efficacy Scale for Children Negative Emotions Subscale, *PSS* Perceived Stress Scale, *SSRQ* Short Version of the Self-Regulation Questionnaire, *ACSI-28* Athletic Coping Skills Inventory, *BAI* Beck Anxiety Inventory, *DASS-21* Depression, Anxiety, and Stress Scales, *SLWS* Satisfaction with Life Scale, *FFMQ* Five Facet Mindfulness Questionnaire, *AAQ-II* Acceptance and Action Questionnaire-II, *CCAPS-62* Counselling Centre Assessment of Psychological Symptoms-62, *DERS* Difficulties with Emotion Regulation Scale, *MAAS* Mindful Attention Awareness Scale, *A-LIT* Anxiety Literacy Questionnaire, *D-LIT* Depression Literacy Questionnaire, *MHLS* Mental Health Literacy Scale, *K-6* Kessler-6, *PCPH* Parental confidence to provide help, *GHSQ* General help-seeking questionnaire, *PSSN* Parent social support network in the sport club environment, *CSE* Coping Self-Efficacy Scale, *FORQ* Fortitude Questionnaire, *AFM-2* Affectometer-2, *PCHB* Previous contact and helping behaviour, *OMHE* Own mental health experience, *IPH* Intentions to provide help, *ISH* Intentions to seek help, *CPH* Confidence to provide help, *POMS* Profile of mood states, *GHQ-12* General Health Questionnaire, *GSES* Generalized Self-Efficacy Scale, *PCS* Perceived Competence Scale, *CDRS* Connor-Davison Resilience Scale, *SDS* Social Distance Scale, *MDSPSS* Multidimensional Scale of Perceived Social Support, *IB* Implicit beliefs, *MHC* Keyes’ Mental Health Continuum, *SCL-90-R* Global Severity Index of the General Psychiatric Symptoms-90-Revised, *AHSQ* Actual help-seeking, *DSS* Depression Stigma Scale, *GASS* Generalised Anxiety Stigma Scale, *?1* No name given to measure with questions around mental health recognition, knowledge and confidence, *?2* No name given to customised measure around attitudes and recognition of depression in clinical scenario, *TMS* Toronto Mindfulness Scale, *STAI* State and Trait Anxiety Inventory, *PANAS* Positive and Negative Affect Schedule, *BRUMS* Brunel Mood Scale, *?3* No name given to measure with questions around mental health confidence to help, *SGMT* Sport-general mental toughness, *RSMT* Referee-specific mental toughness, *SCQ* Song And Hattie Self-Concept Questionnaire, *MHRES* Mental Health Referral Efficacy Scale, *MHRK* Mental Health Referral Knowledge Scale

### Effects on referral efficacy/confidence to help someone with a mental health problem

Confidence to provide help or to successfully refer an individual suffering from a mental health issue was increased in six studies [18, 52, 59, 60, 65, 66]; however, each of these studies deployed measurement tools that have not been psychometrically validated. One study observed significant positive changes for mental health referral efficacy [69] and utilised a validated scale. One study [40] did not observe any significant change in confidence to provide help.

### Effects on help-seeking intentions and behaviour

Three studies [8, 40, 60] observed an increase in intentions to offer help to those with a mental health problem, although in the Liddle et al. study, intentions were not shown to be sustained at a later follow-up period. While Gulliver et al. [63] did not see improvements in intentions to seek help for oneself, two studies reported personal help-seeking improvements [18, 46]. In one study, participants favoured formal help [52] while in another informal help was preferred [57]. In concordance with the findings in the original review, actual behaviour change was not reported in any of the studies.

### Well-being and additional outcomes

Sixteen studies reported improvements in at least one well-being outcome, with six enhanced overall mental health and well-being, six leading to a decrease in stress and four reducing symptoms of anxiety. In contrast, null effects were reported for distress levels [18, 57, 58] well-being and resilience [8] and depression and quality of life outcomes [50]. Two of these studies did not report statistical tests for significance or effect sizes, thus, restricting the interpretation of findings [62, 68]. Other outcomes that were assessed showed improvements, such as emotional intelligence [45], coping [50, 53] and mindful awareness [54, 70], mental toughness [72], relationship domains [62] and substance abuse [62], but were not confirmed with statistical tests for significance.

### Risk of bias assessment

Risk of bias assessment for the two randomised studies is presented in Table 4. The two studies using randomisation methods demonstrated a low [63] and unclear [69] risk of bias. There was no high risk of bias scored for any of the domains across the two studies. Information was not provided on selection, performance and detection bias in [69], giving the design an overall judgement decision as unclear. Across the studies, bias was mixed for random sequence generation, allocation concealment and blinding of participants with [63] scoring low on those domains and [69] scoring unclear. Collectively, bias was unclear for blinding of outcome assessors and both demonstrated a low risk of bias for (a) missing data, (b) selective reporting and (c) other biases.

**Table 4:** Risk of bias for randomised studies using Cochrane risk of bias tool

Study	Random sequence generation	Allocation concealment	Blinding of participants and personnel	Blinding of outcome assessment	Incomplete outcome data	Selective reporting	Other bias	Summary
Ajilchi et al. (2019) [45]	^c^Random draw	^a^Randomisation conducted by an independent party unconnected to project	^c^Study was non-blinded	^c^Study was non-blinded	^a^Each participant completed the intervention	^a^All prespecified outcomes were reported	^b^Authors transparent throughout. Small sample that is not diverse	High risk of bias for this study. Three domains showed a high risk of bias due to high risk of selection, detection and performance bias
Donohue et al. (2018) [47]	^a^Urn randomisation	^b^Unclear who performed randomisation	^b^No measures described to blind participants to intervention	^a^Assessors from clinic that operated independently from intervention programmes. No blinds assessed to be broken.	^a^Analyses adjusted for data being missing at random	^a^All outcome measure effects were reported, along with effect sizes for each group	^a^Study limitations addressed and transparency ensured throughout	Low risk of bias for this study. Two domains were unclear but they were unlikely to have had major bearing on results
Glass et al. (2019) [51]	^a^Stratified random sampling to ensure comparable groups	^b^Unclear who performed randomisation	^b^Participants were asked not to discuss details of intervention but it is possible discussion took place as participants were students at same university	^b^Unclear whether assessors had knowledge of treatment groups when assessing effects	^c^High attrition rate leaves data susceptible to attrition bias	^a^All outcome measure effects were reported, along with effect sizes for each group	^a^Study limitations were highlighted	Unclear risk of bias for this study. Aspects of selection, performance and detection bias were unclear. High risk of attrition bias due to nature of mindfulness intervention
Gross et al. (2018) [44]	^c^Attempt was made to use random selection but due to time constraints it was not employed, decision was taken to use one team.	^b^Unclear who performed randomisation into the two intervention groups	^b^Participants were from the same team so there was potential for discussion about details of intervention	^b^Unclear whether assessors had knowledge of treatment groups when assessing effects	^a^Attrition and losses to follow-up were disclosed and reasons provided	^a^All prespecified outcomes were reported	^b^Potential for allegiance effects influencing results as one of the groups was led by an author of the study but the study showed that therapeutic rapport did not have a significant effect	Unclear risk of bias for this study. Lack of randomisation raises prospect of selection bias but overall the process was transparent
Gulliver et al. (2012) [63]	^a^Automated computer system used	^a^Conditions allocated by researchers not involved in day-to-day management	^a^Described method used to reduce likelihood of participant knowledge of intervention	^b^Unclear whether assessors had knowledge of treatment groups when assessing effects	^a^Analyses adjusted for data being missing at random	^a^All outcome measure effects were reported, along with effect sizes for each group	^a^Study limitations were addressed and caution is urged when interpreting significant effects	Low risk of bias for this study. One domain (blinding of outcome assessors) was unclear but it is unlikely if that influenced the results given the online format of the intervention and data collection
Liddle et al. (2019) [40]	^a^Randomisation occurred using a random number generator	^a^Randomisation conducted by an independent researcher not involved in intervention or data analysis	^a^Participants not informed of allocated condition	^b^Unclear whether assessors had knowledge of treatment groups when assessing effects	^a^Analyses adjusted for data being missing at random	^a^All prespecified outcomes were reported	^a^Authors were transparent about each stage of the intervention design	Low risk of bias for this study. One domain (blinding of outcome assessors) was unclear but it is unlikely to have significant impact on results
Sekizaki et al. (2019) [55]	^a^Randomisation was performed using each student’s school number	^b^Unclear who performed randomisation	^c^Study was non-blinded and in the same school there was risk for sharing of information between groups	^c^Study non-blinded, potential for detection bias	^a^Each participant completed the intervention	^a^All prespecified outcomes were reported	^a^Study limitations were addressed and authors urged caution over the generalizability of the findings	Moderate risk of bias for this study. Selection, attrition and reporting bias risk was low. Risk of performance and detection bias was high due to no blinding.
Van Raalte et al. (2015) [69]	^b^Method not disclosed	^b^Unclear who performed randomisation	^b^Unclear if participants were or were not blinded to their intervention	^b^Unclear whether assessors had knowledge of treatment groups when assessing effects	^a^Analyses adjusted for data being missing at random	^a^All outcome measure effects were reported, along with effect sizes for each group	^a^Authors were transparent about each stage of the intervention design	Unclear risk of bias for this study. Information on selection, performance and detection bias was not disclosed, though attrition and reporting bias was low
Summary of bias across studies	Random sequence generation was performed in each study bar one. One study did not disclose method	Methods of allocation were largely unclear except for three studies where risk of bias was low	Blinding of participants was mixed, 4 studies were unclear while two had high risk and two low	The risk of bias was mixed, 5 studies were unclear while two had high risk and one low for blinding the assessors’ knowledge	7 of 8 studies displayed low risk of bias for controlling missing data, one study was high	There was a low risk of bias across the studies for reporting outcomes	Transparency was ensured by each of the studies, resulting in a low risk of bias for 6 studies and 2 unclear	Risk of selection, performance and detection bias findings were mixed. The risk for attrition and reporting bias was low with transparency maintained throughout each of the studies

^a^Low risk of bias

^b^Unclear risk of bias

^c^High risk of bias

Risk-of-bias assessment for each of the randomised controlled trials is presented in Table 4. Three studies [40, 47, 63] demonstrated a low risk of bias. For three of the studies [44, 51, 69] the risk of bias was deemed to be unclear as there was insufficient information provided on selection, performance and detection bias. One study was deemed to be of a moderate risk of bias [55] as a lack of blinding raised the risk of performance and detection bias. An invalid method of random sequence generation and non-blinding forced one study [45] to be adjudged as high risk of bias. Collectively, the risk of selection, performance and detection bias findings were mixed. The risk of attrition and reporting bias was generally low.

Risk-of-bias for each of the non-randomised studies is depicted in Table 5. Twelve studies were judged to have a weak study quality. Nine studies were characterised as being of moderate quality as a result of having one domain that was considered to be weak. A lack of clarity on blinding of assessors and participants was weak in 5 of those moderate quality studies. High attrition rate or unclear disclosure of dropout rate accounted for the weak domain in the remainder of the moderate quality studies (*n* = 4). Each of the non-randomised studies was of strong or moderate quality in controlling for selection bias, study design and data collection methods. A mix of quality was seen in the confounder and withdrawal domains, with nine and seven weak studies respectively. Sixteen of the non-randomised studies were of weak quality for blinding while the remaining five had moderate ratings.

**Table 5:** Risk of bias for non-randomised studies using the Quality Assessment Tool for Quantitative Studies (QATSQ) tool

Study	Selection bias	Study design	Confounders	Blinding	Data collection methods	Withdrawals and dropouts	Summary
Bapat et al. (2009) [59]	2	2	3	3	3	3	
	Participants are very likely to be representative Cannot tell the percentage of participants who agreed	Study is designated as a cohort analytic study	There were gender and age differences that may have influenced the outcomes between participants and these were not controlled for in analysis	Outcome assessors knew intervention status, and blinding of participants to research question is not described	The validity and reliability of the instruments are not described	Withdrawals and dropouts were not described	Weak quality: as this study scored four weak ratings, the overall judgement is weak quality
Breslin et al. (2017) [8, 60]	2	1	1	2	1	3	
	Participants are very likely to be representative Cannot tell percentage of participants who agreed	Study is designated as a controlled clinical trial	Confounders (gender, sport type) were similar across control and intervention groups	Cannot tell if outcome assessors were aware of intervention status and cannot tell if intervention participants were aware of research question	Tools were shown to be valid and reliable	Cannot tell if there were withdrawals or dropouts	Moderate quality: As this study scored one weak rating the overall judgement is moderate quality
Breslin et al. (2018)	1	1	2	2	1	3	
	Participants are very likely to be representative. All participants agreed to participate	Study is designated as a controlled clinical trial	Age differences between groups may have acted as confounder. Other significant demographic differences were controlled for	Cannot tell if outcome assessors were aware of intervention status and cannot tell if intervention participants were aware of research question	Tools were shown to be valid and reliable	Significant drop out rate was described and reasons provided	Moderate quality; As this study scored one weak rating the overall judgement is moderate quality
Chow et al. (2020) [46]	1	2	2	3	1	1	
	Participants are very likely to be representative. All participants agreed to participate	Study is designated as a cohort analytic study	No significant baseline differences between those who had mental health experience and those who had not therefore groups were combined for primary analysis	Outcome assessors knew intervention status and blinding of participants to research question is not described	Tools were shown to be valid and reliable	100% completion rate at follow-up	Moderate quality; as this study scored one weak rating the overall judgement is moderate quality
Donohue et al. (2015) [62]	1	2	3	3	1	2	
	Participants are very likely to be representative All participants agreed to participate	Study is designated as a cohort analytic study	There were gender, ethnic and age differences that may have influenced the direction of result. These were not controlled for in the analysis	Outcome assessors knew intervention status, and the participants knew intended outcome of the research (i.e. developing intervention)	The validity and reliability of the instruments is described	There was a 70% follow-up rate from those that consented and completed the intervention	Weak quality: as this study scored two weak ratings, the overall judgement is weak quality
Dowell et al. (2020) [48]	2	2	3	3	2	3	
	Participants are somewhat likely to be representative, fee required may influence sample. All participants agreed to participate	Study is designated as a cohort analytic study	The requirement to control confounders was alluded to but the rationale behind adjustment was not sufficiently transparent	Outcome assessors knew intervention status and blinding of participants to research question is not described	Some tools were shown to be valid and reliable, low internal consistency was observed for measuring conduct problems	Less than 50% of initial sample completed intervention	Weak quality; as this study scored three weak ratings the overall judgement is weak quality
Dubuc-Charbonneau and Durand-Bush (2015) [49]	1	2	3	3	1	1	
	Participants are very likely to be representative. All participants agreed to participate	Study is designated as a cohort analytic study	Confounding variables were not discussed	Outcome assessors knew intervention status and blinding of participants to research question is not described	Tools were shown to be valid and reliable	100% completion rate at follow-up	Weak quality; as this study scored two weak ratings, the overall judgement is weak quality
Fogaca (2019) [50]	2	1	3	3	1	2	
	Participants are somewhat likely to be representative. Risk of selection bias by removal of one team from intervention group data. Above 80% of participants agreed to participate	Study is designated as a controlled clinical trial	Study showed that there were no significant differences between intervention and control for mental health measures pre-test with the exception of depression, as a result the outlying team was removed from the data. No discussion of demographic differences (potential confounders) between intervention and control	Outcome assessor knew intervention status and blinding of participants to research question is not described	Tools were shown to be valid and reliable	60–79% completion rate	Weak quality; as this study scored two weak ratings, the overall judgement is weak quality
Hurley et al. (2018) [18]	1	1	2	3	1	1	
	Participants are very likely to be representative. All participants agreed to participate	Study is designated as a controlled clinical trial	The study deploys a ‘matched’ control group to attempt to control for confounding variables but no mention of whether this holds true	Outcome assessors knew intervention status and blinding of participants to research question is not described	Tools were shown to be valid and reliable	> 80% completion rate at follow-up	Moderate quality; as this study scored one weak rating, the overall judgement is moderate quality
Hurley et al. (2020) [52]	1	1	1	2	1	3	
	Participants are very likely to be representative. All participants agreed to participate	Study is designated as a controlled clinical trial	Matched control trial to account for confounding variables. Covariates are adjusted for	Cannot tell if outcome assessors were aware of intervention status and blinding of participants to research question is not described	Tools were shown to be valid and reliable	Retention of participants was low particularly in the control group	Moderate quality; as this study scored one weak rating, the overall judgement is moderate quality
Laureano et al. (2014) [53]	2	1	2	3	2	1	
	Participants are somewhat likely to be representative. All participants agreed to participate	Study is designated as a controlled clinical trial	Study corrected for pre-test differences. However, extraneous variables impacting cannot be ruled out	Outcome assessors knew intervention status and blinding of participants to research question is not described	Some tools were shown to be valid and reliable, FORQ results should be treated tentatively due to low internal consistency	100% completion rate at follow-up of intervention and control groups	Moderate quality; as this study scored one weak rating, the overall judgement is moderate quality
Longshore and Sachs (2015) [64]	1	1	1	3	3	1	
	Participants are very likely to be representative Above 80% of participants agreed to participate	Study is designated as a controlled clinical trial.	No significant differences were found between the groups before the intervention	Outcome assessors knew intervention status, and the participants knew intended outcome of the research (i.e. benefits of mindfulness)	The validity and reliability of the instruments is not described	There was a > 80% follow-up rate from those that consented and completed the intervention	Weak quality: as this study scored two weak ratings, the overall judgement is weak quality
Mohammed et al. (2018) [54]	2	1	1	3	2	1	
	Participants are somewhat likely to be representative. >80% of participants agreed to participate	Study is designated as a controlled clinical trial	Confounders were similar across intervention and control group	Outcome assessors knew intervention status and participants were not blinded to research question	The tools deployed displayed varied levels of validity and reliability	> 80% completion rate at follow-up	Moderate quality; as this study scored one weak rating, the overall judgement is moderate quality
Pierce et al. (2010) [65]	2	2	3	3	3	2	
	Participants are very likely to be representative Cannot tell the percentage of participants who agreed	Study is designated as a cohort analytic study	There were age and education differences that may have influenced the direction of result these were not controlled for in the analysis	Outcome assessors knew intervention status, and the participants knew intended outcome of the research (i.e. respond to mental health problems)	The validity and reliability of the instruments is not described	There was a 66% follow-up rate from those that consented and completed the intervention	Weak quality: as this study scored three weak ratings, the overall judgement is weak quality
Sebbens et al. (2016) [66]	1	1	1	3	3	1	
	Participants are very likely to be representative Above 80% of participants agreed to participate	Study is designated as a controlled clinical trial	No significant demographic differences were found between the groups before the intervention	Outcome assessors knew intervention status, and blinding of participants to research question is not described	The validity and reliability of the instruments is not described	There was a > 80% follow-up rate from those that consented and completed the intervention	Weak quality: As this study scored two weak ratings, the overall judgement is weak quality
Slack et al. (2015) [67]	1	2	3	3	3	1	
	Participants are very likely to be representative Above 80% of participants agreed to participate	Study is designated as a cohort analytic study	Confounding variables were not discussed	Outcome assessors knew intervention status, and blinding of participants to research question is not described	While one measure was referenced as valid and reliable, no information was reported on validity and reliability of another measure (RSMT)	There was a > 80% follow-up rate from those that consented and completed the intervention	Weak quality: As this study scored three weak ratings, the overall judgement is weak quality
Shannon et al. (2019) [56]	1	1	1	3	1	2	
	Participants are very likely to be representative. All participants agreed to participate	Study is designated as a controlled clinical trial	Baseline measurements indicated that there were no significant differences between control and intervention group for study outcomes or gender. Age was significantly different but analysis showed it did not have a significant effect on outcomes	Outcome assessors knew intervention status and blinding of participants to research question is not described	Tools were shown to be valid and reliable	There is no information provided about withdrawals or dropouts but Little’s MCAR analyses revealed data was missing at random and the expectation maximisation algorithm was used to estimate missing values	Moderate quality; as this study scored one weak rating, the overall judgement is moderate quality
Tester et al. (1999) [68]	2	2	3	2	1	3	
	Participants are very likely to be representative Cannot tell the percentage of participants who agreed	Study is designated as a cohort analytic study	Confounding variables were not discussed	Cannot tell if outcome assessors were aware of intervention status Cannot tell if intervention participants were aware of research question	Tools were referenced as valid and reliable	Cannot tell if there were withdrawals or dropouts	Weak quality: As this study scored two weak ratings, the overall judgement is weak quality
Vella et al. (2020) [57]	1	1	1	2	2	3	
	Participants are very likely to be representative. All participants agreed to participate	Study is designated as a controlled clinical trial	Matched control to account for confounding variables. Baseline differences are highlighted and adjusted for	Cannot tell if outcome assessors were aware of intervention status and blinding of participants to research question is not described	Majority of tools were shown to be valid and reliable except low internal consistency for implicit beliefs scale	A small proportion of participants completed the entire intervention per protocol	Moderate quality; as this study scored one weak rating, the overall judgement is moderate quality
Vidic et al. (2018) [58]	1	2	3	3	1	3	
	Participants are very likely to be representative. All participants agreed to participate	Study is a cohort design	Did not control for confounding variables	Outcome assessor knew intervention status and blinding of participants to research question is not described	Tool used was shown to be valid and reliable	There is no information provided about withdrawals or dropouts	Weak quality; as this study scored more than two weak ratings, the overall judgement is weak quality
Summary of bias across the studies	Twelve studies were of strong quality and controlled for selection bias, the remaining 8 were of moderate quality	Eleven studies were of strong quality for study design and the remaining 9 were of moderate quality	There was a mixture of strong (*n* = 7), weak (*n* = 9) and moderate (*n* = 4) information provided on confounders	Fifteen of the non-randomised studies were of weak quality for blinding participants and outcome assessors. 5 were of moderate quality	Eleven of the non-randomised studies were of strong quality and referenced adequate reliability and validity for outcome measures, while 9 studies used tools of varied validity	There was a mixture of strong (*n* = 8), weak (*n* = 8) and moderate (*n* = 4) for the researchers’ disclosure of follow-up rates and dropouts	Nine studies were deemed to be of moderate quality and 11 were of weak quality

1 = strong; 2 = moderate; 3 = weak

### Outcome measure validity assessment

Sixteen studies were assessed to have acceptable outcome measures as their scales used had adequate internal consistency or referenced studies that had validated the scales used previously. Eight studies used some scales that had displayed adequate validity and reliability, while others deployed tools that had not met the predefined criteria. Five studies were deemed to be unacceptable.

## Discussion

This updated systematic review analysed the recent proliferation of published studies in the field of mental health and sport, thus providing a more inclusive and contemporary reflection of the evidence base for those involved in the design, implementation and receival of mental health awareness programmes. While the previous review articulated the increasing recognition that athletes, coaches and officials in sport settings can be vulnerable to mental health problems [1, 73, 74], the identification of eighteen further studies in this updated review mirrors the wider exposure of sport and mental health evident in the media and wider public health agenda [75]. Overall, support was maintained for improving mental health knowledge and help-seeking among coaches, athletes and officials, with extensions to multicomponent programmes that included parents, athletes and coaches. However, and despite some improvements in methodological quality of the field, issues persist such as a lack of theoretical input into both programme design and analyses, and lack of long-term follow-up data collection periods.

### Effects of studies on awareness outcomes

The studies that examined the effects of intervention programmes on mental health stigma produced mixed outcomes. For instance, one study revealed a reduction in stigma surrounding anxiety, however depression stigma remained unchanged [63], another study showed a reduction in stigma about their own mental health help-seeking but no significant effect was shown for public stigmatisation [46]. Liddle et al. [40] showed that stigmatising attitudes were reduced and sustained at follow-up; however, this study along with three other studies [57, 59, 60] examining stigma did not include effect sizes, which makes it difficult to ascertain the extent of intervention impact on mental health stigma.

Eleven of the twelve studies within the review that examined mental health knowledge demonstrated an improvement in at least one aspect of mental health knowledge. However, aside from the six studies that used the A-LIT [63] and D-LIT [71] questionnaires, generalisability of the other studies is difficult due to substantial heterogeneity in measurement tools used. That being said, there is definite potential to improve mental health knowledge in sport participants through awareness interventions, going forward, efforts to increase mental health literacy could be more clearly demonstrated if equivalent measuring tools were deployed across studies.

Confidence in oneself to help or successfully refer someone with a mental health problem was improved in seven studies, only one study showed no significant effect [40]. Of methodological interest, only one study deployed a randomisation procedure, reported effect sizes, maintained longitudinal effects and provided evidence for validity of outcome measures [69]. Three studies showed an increase in intentions to offer help to a person experiencing mental health problems [8, 40, 60]; however, the intentions to support in Liddle et al. [40] were not sustained at follow-up. Further research is required to identify the favoured form of help-seeking for sport participants as one study [52] showed formal help-seeking was preferred, while another [57] indicated that help-seeking from informal sources was favoured. Similar to the previous review conclusion behaviour change was not reported in any of the studies, highlighting an area that requires further design and measurement consideration.

One of the 18 additional studies [56] (a mindfulness-based intervention) deployed a behaviour change model: Self-Determination Theory [76]. The study findings supported competence-promoting processes within the intervention, indicating that engaging with mindfulness practices can confer perceptions of competence in mental health self-management with subsequent indirect effects on stress regulation, mindfulness and overall well-being [56]. While the effect sizes were small, the findings showed that future programme design could benefit from the inclusion of behaviour change models.

To assess the long-term effects of the programmes on mental health and wellbeing improvement, the inclusion of post-intervention follow-up data is crucial. While seventeen studies displayed improvements in some aspect of well-being, only five studies included post-intervention follow-up [40, 44, 47, 51, 62]. Each of these longitudinal studies evidenced sustained improvements in depression, generalised anxiety and distress at follow-up.

### Methodological quality of studies

Close inspection of the studies indicates various design limitations, these need to be overcome for future development of programmes. The previous review identified a lack of overall methodological rigour and a high risk of bias among the included studies. The authors described a need for further ‘well-designed controlled trials’ [60]. While some flaws remain in a number of the 18 additional articles included in the updated review, a trend toward higher quality studies with a lower risk of bias can be observed. Five of the ten studies within the previous review did not include a control group (50%), the percentage of studies without a control group in the additional 18 articles was greatly reduced to 3 studies [46, 48, 58]. Two studies within the previous review had a randomised control trial design; the total in the updated review is now eight. However, the generalisability of several of the studies is hindered by the small sample sizes included within the review; thus, the long-term significance of these studies remains unclear. Three studies did not report gender which prevents the review from providing any further insight into gender effects on help-seeking behaviours [77, 78].

Seven of the previous eight non-randomised studies were of weak quality and one moderate. Eight of the additional twelve non-randomised studies were of moderate quality and four were weak. Collectively, three of the randomised controlled trials displayed a low risk of bias, four were unclear and one was of high risk. Risk of selection, performance and detection bias was high in several studies due to a lack of randomisation and blinding measures in certain studies. Risk of attrition and reporting bias was low. While there were some signs of improvement in meeting the predefined criteria for acceptable psychometric measurement validity, significant heterogeneity remained present. The majority of referral efficacy tools lacked validation; therefore, it is not possible to take great confidence from the effects reported.

Overall, it is clear that a higher proportion of studies have adhered to methodological guidance in the design and reporting of interventions as was advised in the previous review. Examples from the current review show that research deemed to be of weak quality, deploying a ‘pre-post intervention’ design, with no or short follow-up periods [62], tend to be extended in more recent times to have greater methodological rigour, utilised a randomised control trial design and included 8 month follow-up periods, with low risk of bias [47]. These developments are indicative of an area of research that is improving as the recognition of mental health in sport grows internationally.

### Intervention delivery methods

The content of each of the programmes varied and the attendees involved were from a variety of backgrounds within a sport setting, i.e. elite athletes, coaches, club leaders, student athletes, officials, parents and those athletes who had been referred after reporting substance misuse (see Table 2). Therefore, future reviews may want to consider limiting the search to a particular group only (i.e. athletes, coaches, officials, parents or athletes considered to be at high risk).

Similarly, the frequency and duration of sessions for each programme varied greatly, for instance: an 8-h programme across three group sessions [59]; 12 separate group sessions each with a different topic; 12 individual one to one sessions; a programme that lasted 1.5 h initially then completing a home programme for 6 weeks [64]; to a 45-min one-off workshop [40]. Programmes were delivered online or delivered in groups by trained facilitators. Determining the most effective delivery method (i.e. online, one to one, in groups) and intervention duration and frequency is not possible from the current review, but could be considered a screening variable for future reviews.

### Limitations and recommendations

The review was limited to studies published in English which could perhaps have forced the exclusion of data from certain parts of the world, narrowing the generalisability of the review.

As has been discussed, in spite of an overall trend toward a higher quality of research, there remain inconsistencies in the outcome measures. In addition to small sample sizes, these limitations negatively impact upon the long-term significance of study findings. This review is limited by its exclusion of grey literature. When the research was proposed, it planned for a full search of the grey literature, searching for programmes published by the government, sporting governing bodies such as the NCAA and national public health agencies. The decision was made to exclude grey literature due to the time constraints. Further research could expand on this update by incorporating insights from grey literature. Excluding any form of potentially valuable information goes against the nature of systematic reviews which aim to summarise the findings of all relevant studies [79]. Furthermore, qualitative data was not included as it would be difficult to assume a level of generalisability between quantitative and qualitative findings. Individual case study work of applied sport psychologists was identified but excluded from the review, and thus, the review may miss out on valuable expert insights.

There are several recommendations from the studies included in this review. While one study incorporated theory of behaviour change in design and evaluation of the intervention, the remaining studies did not. Application of behaviour change modalities can allow researchers to identify the motivational factors that influence the decision-making process to seeking help. With a collection of studies using theory, interventions can then be tailored to target these factors and in turn influence behaviour [80]. The inclusion of some more established theories of behaviour change have associated valid and reliable measurement tools [10], the incorporation of these tools would enhance future mental health awareness programme evaluation.

Further, the mental health of sports officials is a pressing concern. The lack of research aimed toward officials within this review provides further evidence for the ‘Call to action: the need for a mental health research agenda for sports match officials’ [4]. It is imperative that research is carried out within officiating to allow for evidence-based mental health interventions to be implemented among officials.

Initiatives that target multiple levels of influence (e.g peers, coaches, environment, systemic influences) have great potential to be more effective than more narrow research. Studies within this review have shown the potential benefits of targeting parents to create a supportive environment for their participating child [18, 52, 57]. Furthermore, coaches who manage stress effectively have been shown to be better equipped to support athletes in dealing with stress [81, 82]. Providing training for coaches in mental health awareness could contribute to changing the culture of help-seeking in sport, providing an environment in which athletes feel more comfortable to seek mental health support and services [66]. As has been alluded to, more longitudinal studies are required to assess the long-term impacts of the included interventions. Future research should seek to attain follow-up information where possible as this would be useful in future planning of intervention content.

## Conclusions

This updated systematic review reaffirms the benefits and the urgent need for evidence and theory-based intervention programmes designed to increase mental health awareness to aid prevention and provide support for athletes, coaches, officials and parents who are suffering from a mental health problem. The contribution of the updated review has deduced that there has been a trend toward research of higher methodological quality and reduced risk of bias in the intervening time since the previous review was published. Therefore, greater value can be placed on the findings within studies in this updated review. However, there remains room for improvement in research quality. For instance, future longitudinal studies are required with larger sample sizes, randomisation to groups should be double-blinded and outcomes should be measured with externally validated measurement tools. Programme designers would benefit greatly from considering grounding programme content in relevant behaviour change theory to more effectively tailor programmes to the motivational needs of participants. To conclude, the findings within this review can aid the development of sport-specific programmes to increase the mental health awareness and well-being of the vulnerable, underserved sporting population and can contribute to reducing the overall burden of global mental health.

## Supplementary Information

**Additional file 1.** PRISMA 2020 Checklist.

**Additional file 2.** PSYCINFO. Advanced search- English Only. Apply related words, apply related subjects.

## Data Availability

Data generated during this study are included in Tables 1, 2, 3, 4 and 5 and Figs. 1 and 2.

## Abbreviations

MRC: Medical Research Council
PRISMA: Preferred Reporting Items for Systematic Reviews and Meta-Analyses
PROSPERO: International prospective register of systematic reviews
QATSQ: Quality Assessment Tool for Quantitative Studies
SNI: Sport Northern Ireland
UK: United Kingdom
USA: United States of America
WHO: World Health Organisation
