# Supplementary material for: An updated systematic review of interventions to increase awareness of mental health and well-being in athletes, coaches, officials and parents
Source: Syst Rev. 2022 May 19;11:99. doi: 10.1186/s13643-022-01932-5 (PMC9118780; doi:10.1186/s13643-022-01932-5)
Supplement: Supplementary file 2 — Additional file 2. PSYCINFO. Advanced search- English Only. Apply related words, apply related subjects. [file 13643_2022_1932_MOESM2_ESM.docx]

Exp Sports/- 63,547

1. Sport$. Ab,ti- 10,684
2. 1 or 2- 63,547
3. Exp teachers/- 243,937
4. (Teacher$ or Leader$ or instructor$ or player$ or member$ or participant$ or coach$ or official$ or parent$) ab-956,328
5. (Teacher$ or Leader$ or instructor$ or player$ or member$ or participant$ or coach$ or official$ or parent$)ti-135,944
6. 5 and 6- 113,973
7. 4 or 7- 303,564
8. 3 and 8-7,279
9. Sports coaching/- 2,207
10. Exp Athletes/-19,346
11. Athlete$.ab,ti.- 5110
12. 10 or 11 or 12-20,505
13. 9 Or 13-24,723
14. Schools/ or colleges/ or high schools/- 2,686,104
15. (Sport$ adj3 (organi#ation$ or club$ or governing bod$ or cent$ or school$ or setting$)).ab- 510,221
16. (Sport$ adj3 (organi#ation$ or club$ or governing bod$ or cent$ or school$ or setting$)).ti- 142,460
17. (Sport$ adj3 (organi#ation$ or club$ or governing bod$ or cent$ or school$ or setting$)).ab,ti.-106,943
18. 15 or 18- 2,691,024
19. 14 and 19- 19,596
20. Mental health/-558,827
21. Well being/-229,548
22. (mental$ adj3 (health or wellbeing or well being or well-being or wellness or ill$))ab.-172,506
23. TI mental$ adj3 AND TI health OR TI wellbeing OR TI well-being OR TI wellness OR TI ill- 50,180
24. Anxiety/-251,930
25. “Depression(emotion)”/-23,373
26. (anxiety or depress$).ab-179,900
27. (anxiety or depress$).ti-51,831
28. (mental$ adj3 (health or wellbeing or well being or well-being or wellness or ill$)).ab.ti-43,951
29. (anxiety or depress$).ab,ti.- 46,067
30. 21 or 22 or 29 or 25 or 26 or 30- 925,800
31. 20&31-3314
32. Internet/- 56,746
33. Websites/- 19,040
34. (internet or online or website$ or web site$ or web based).af.-954
35. 33 or 34 or 35- 68,062
36. 19 or 36- 2,704,168 overall setting
37. 31 and 36-17,362
38. 8 or 13-320,804
39. 3 and 31 and 37 and 39-3057
40. (40)ab or ti- 483
41. (40) ab and ti- 14
42. 41(2015-20)-182
43. Internet etc all fields-136822
44. 32 OR 33 OR 43- 136822
45. 18 OR 44- 2735847
46. 3 AND 30 AND 38 AND 46-3110
47. 46 ab or ti-1176
48. 47 AND ab intervention*-192
49. 47 AND ti intervention*-49
50. 47 AND ab train*-218
51. 47 AND ab program*-188
52. 47 AND ti program*-43
53. 47 AND ti train*-43
54. 48-53 OR- 451
55. 54 2016-2020-175
